# Drug resistance and tumor heterogeneity: cells and ensembles

**DOI:** 10.1007/s12551-025-01320-y

**Published:** 2025-05-31

**Authors:** Ruth Nussinov, Bengi Ruken Yavuz, Hyunbum Jang

**Affiliations:** 1 Computational Structural Biology Section, Frederick National Laboratory for Cancer Research, Frederick, MD 21702 USA; 2 Department of Human Molecular Genetics and Biochemistry, Sackler School of Medicine, Tel Aviv University, 69978 Tel Aviv, Israel; 3 Cancer Innovation Laboratory, National Cancer Institute, Frederick, MD 21702 USA

**Keywords:** Drug resistance, Cancer, Dynamic conformational ensembles, Heterogeneity, Allostery, Allosteric

## Abstract

The population of cells that make up a tumor, and of their biomolecular conformational ensembles, are heterogeneous at all levels, genetic, epigenetic, and phenotypic. At the cellular level, tumor heterogeneity was described as the “Rosetta Stone of therapy resistance.” At the genetic level, tumors consist of divergent tumor (sub)clones. At the phenotypic level, their observed function, clinical attributes, and response to drugs vary. We suggest that the behavior and properties of populations of cells—and of populations of conformational states—are intrinsically connected. This is important. Considering the tumor’s disruption of normal cellular processes clarifies why it is crucial to understand the ins and outs of its mechanistic molecular foundation. In reality, the propensities of the tumor’s conformational states underly the proliferative potential of its cell populations. These propensities are determined by expression levels, driver mutations, and the tumor cells environment, collectively transforming tumor cells behavior and crucially, drug resistance. We suggest that propensities of the conformations, across the tumor space and over time, shape tumor heterogeneity, and cell plasticity. The conformational states that are preferentially visited can be viewed as phenotypic determinants, and their mutations and altered expression work by allosterically shifting the relative propensities, thus the cell phenotype. Physics (and chemistry) inspire the notion that living things must conform to fundamental laws of science, like dynamic landscapes. Dynamic conformational propensities are at the core of cell life, including tumor cells; their heterogeneity is the formidable, unmet drug resistance challenge.

## Introduction

Awe-inspiring advances are being made in drug discovery. The list is breathtaking. These include innovative, landmark single-molecule drug discovery successes and drug combinations. Yet, targeting tumor heterogeneity—the core of the drug resistance—has been lagging. To date, no productive strategy is in sight. There have been efforts to address it, including involving single-cell approaches, for example by characterizing heterogeneous single-cell dose responses (Kinnunen et al. 2024; Tang et al. 2023; Wu et al. 2020) and single-cell RNA-seq data (Fustero-Torre et al. 2021; Li et al. 2024a; Maeser et al. 2024). Despite their efficiencies and innovation, so far, such endeavors did not translate to concrete drugs. A major obstacle is that even single cell characterization falls short in capturing cancer cell heterogeneity. The reason is that heterogeneity exists not only among cells, but *within single cells*. A recent publication defined tumor heterogeneity as the uneven distribution of genetic diversity within tumor cells, both spatially and temporally, which the authors noted, can lead to different responses to drugs within the same tumor lesion (Zhu et al. 2021). Here we pinpoint the “within tumor cell”: the heterogeneity rests in the protein conformational ensembles.

Commonly, heterogeneity is considered across the tumor cell populations. We view it both on the tumor cellular level, and on a more fundamental level, that of protein conformations. Heterogeneity stems from genetic mutations, which alter the distribution of the conformational ensembles. It also emerges from the size of the population of the protein molecules, that is, from overexpression. Overexpression “floods” the cellular network, overspilling through pathway crosslinkages. Overexpression results from, e.g., gene duplication, including super-enhancers, and critically epigenetics. Both genetic and epigenetics scenarios cause conformational heterogeneity. We view the heterogeneous tumor cell populations as largely determined by their heterogeneous conformational ensembles. Heterogeneous conformational ensembles are ensembles of variants of the same protein under different conditions. Since different conformations have distinct preferred binding partners, the propensities of protein conformations determine can predict cell function and phenotypes (Nussinov et al. 2023a; b). Bulk measurements of the single cell provide averaged data. They would struggle to provide detailed distributions.

The importance of *conformational heterogeneity* can be gauged by considering mutation-specificity in drug response. Optimal inhibitors are mutation specific. Precision medicine is based on the ability of anticancer drugs to block specific genetic mutations (Du et al. 2020; Jang et al. 2020; Stallard 2017; To et al. 2022). Mutations do not create new conformations. Instead, they redistribute the conformational ensembles. This challenges technologies that measure bulk single cell response.

Here, we focus on tumor heterogeneity of cell populations, currently the focus of multiple studies, and on conformational ensembles, which are largely at their core, and are influenced by their cellular environment, expression level, and genetics. The challenge is in *how* to address it in drug discovery.

Increasing heterogeneity in drug resistance is akin to dedifferentiation, which has been thought of as “reverse evolution.” Dedifferentiation, a consequence of epigenetic alterations during cancer development, alters the expression levels of the affected proteins, thus signaling, leading to loss of cell-specific functions, thereby increasing cell heterogeneity. However, the heterogeneity may not be a random distribution. As we learnt from early contributions by geneticist Leo Sachs and (statistical) physicist Eytan Domany (Axelsen et al. 2007), in the tumors that they examined, overexpressed genes in brain (astrocytoma and glioblastoma), breast, colon, endometrium, kidney, liver, lung, ovary, prostate, skin, and thyroid, were mostly those that are expressed normally in other tissues. As they noted, “Nearly all of the genes with tissue-selective expression that are overexpressed in cancers showed selective expression in tissues different from the cancers’ tissue of origin. Cancers aberrantly expressing such genes may acquire phenotypic alterations that contribute to cancer cell viability, growth, and metastasis” (Axelsen et al. 2007). In their analysis, melanomas overexpressed the highest number of brain-selective genes, which they suggested may contribute to melanoma metastasis to the brain. These observations could reflect diverse genomic tactics, including gene colocalization, and chromatin dysregulation, pointing to epigenetics. In neurodevelopmental disorders, phenotypic overlaps are common (Jang et al. 2023; Nussinov et al. 2022b, 2023c, 2024 d). In neurological manifestation, it is challenging to define the associated genes. In cancer, identifiable phenotypes could be associated with patterns of expression.

## Heterogeneity, state transitions, and disease

We link biophysics and cancer, connecting single cell biology and genetics of cell populations with the fundamental behavior of conformational ensembles, the entities charged with its regulation (Nussinov et al. 2024 d). Tumor heterogeneity is a crucial case in point. Tumor heterogeneity commonly denotes the variability among tumor cell populations (Caiado et al. 2016; de Bruin et al. 2013; Francis et al. 2014; Gay et al. 2016; Hayford et al. 2021; Hu et al. 2017; Jamal-Hanjani et al. 2017; Lenz et al. 2022; McGranahan and Swanton 2017; Prasetyanti and Medema 2017; Proietto et al. 2023; Stoakes 2024; Welch 2016; Wu et al. 2021; Zhu et al. 2021). Because of its relevance to drug resistance and tumor aggressiveness, the heterogeneity of tumor cell populations has become a key challenge (Dagogo-Jack and Shaw 2018; El-Sayes et al. 2021; Hu et al. 2017; Khatib et al. 2020; Lovly et al. 2016; Sun and Yu 2015; Tammela and Sage 2020; Zhao et al. 2014b; Zhu et al. 2021). Classically, a primary reason for the heterogeneity is expression of oncogenic mutational variants (Beckman and Loeb 2020; Eisenstadt 2016; Gao et al. 2017; Gou et al. 2024; Kim et al. 2016; Lawrence-Paul et al. 2024; Nussinov and Jang 2024; Nussinov et al. 2019b; 2020; Rasnic et al. 2020; Scholl and Frohling 2019; Taha et al. 2021; Wang et al. 2022; Yavuz et al. 2023). Molecularly targeted therapies decimate sensitive mutant tumor cells. However, they also lead to proliferation of minor, rare, resistant cell subclones, increasing the heterogeneity. Overexpression (Albero et al. 2018; Lucchesi et al. 2021; Mohseni et al. 2014; Moriya 2015; Nussinov et al. 2016; Saeki et al. 2023; Tsai and Nussinov 2019) due to gene duplication (Cidado and Park 2012; Karthikeyan et al. 2021), fusion (Chi 2025; Gkountakos et al. 2024; Jin et al. 2022; Kumar-Sinha et al. 2015; Kumar et al. 2024; Latysheva and Babu 2016; Latysheva et al. 2016; Li et al. 2024b; Liu et al. 2023, 2022b; Marsh and Owen 2023; National Cancer Institute 2020; Nussinov et al. 2021; 2022c; Nussinov et al. 2024b; Selvanathan et al. 2015; Taniue and Akimitsu 2021; Tuna et al. 2019), and dysregulated epigenetic modulators (Ansari and Abbas 2022; Banerjee et al. 2024a; Berglund et al. 2021; Cheng et al. 2019; Cristancho and Marsh 2020; Dai et al. 2021; Di Pietro and Good-Jacobson 2018; Giorgi and Del Re 2021; Ilango et al. 2020; Komninou and Richie 2009; Lio et al. 2019; McClellan et al. 2023; Webb et al. 2022), especially common in aggressive and late-stage cancers, abet similar outcome. A 2014 piece (Kiesler 2014) raised the question of “What Is Tumor Heterogeneity?”, postulating that “Understanding tumor heterogeneity may be the next big quest in cancer science.” This has been taken up by scores of papers (e.g., see Aibar et al. 2017; Barkley et al. 2022; Baron et al. 2020; Blise et al. 2022; Chen et al. 2021; Denisenko et al. 2024; Gavish et al. 2023; Gay et al. 2016; Guilliams et al. 2022; Jacquemin et al. 2022; Ji et al. 2020; Kinker et al. 2020; Lee et al. 2020a; Luca et al. 2021; Lugano et al. 2020; Mavrommati et al. 2021; Raghavan et al. 2021; Ravi et al. 2022; Schurch et al. 2020; Zhang et al. 2018)). Because tumor cells can harbor different mutations, it has been challenging to select effective drug regimen, suggesting why the improvement of survival rates of aggressive cancers has been limited (under 20%) despite oncological advances (Siegel et al. 2019). Tumor heterogeneity, especially of cancer stem cells, is a leading cause of cancer progression and treatment failure (Chen et al. 2015; Crucitta et al. 2022; Hayford et al. 2021; Kapoor-Narula and Lenka 2022; Lim and Ma 2019; Marusyk et al. 2020; Prasetyanti and Medema 2017; Safri et al. 2024; Zhang et al. 2022a). Compared to normal stem cells, altered transcriptomes of cancer stem cells have been observed as enriched for functions such as vessel morphogenesis, motility, and metabolism (Gwili et al. 2021), contributing to tumor growth, metastasis, and therapeutic resistance (Ma et al. 2019). Overexpression, which increases the populations of certain proteins, may result in considerably larger heterogeneity. Biologists and clinicians agree that defining oncogenic activating mutations may be a key step in fighting cancer, which is why the immense efforts invested in sequencing cancer genomes, and the highly popular databases housing them. Efforts have also been focusing on revealing cancer-specific overexpression strategies. However, the detailed foundational mechanism underlying it has largely been overlooked. Recently, physics-based views offered innovative perspectives. Robert Vonderheide and Ben Stanger and their coworkers (Kim et al. 2024) offered that epithelial-mesenchymal transitions (EMTs) are the culprit, suggesting that acquired resistance is the product of plasticity rather than the outgrowth of pre-existing mutant subclones. Chandrima Das, John Tainer and Tej Pandita and their collaborators (Das et al. 2024) articulated a “prismatic view of the epigenetic-metabolic regulatory axis in breast cancer therapy resistance,” offering coupling genome-wide analysis with an understanding of metabolic elements, epigenetic reprogramming, and their integration.

In a pioneering work published four decades ago (1984), Elasser suggested that cellular heterogeneity can be thought of as operating entirely within the framework of quantum mechanics (Elsasser 1984). Being unaware of this seminal work at the time, in 2014, Nussinov and Wolynes (2014) noted that even living things must conform to the laws of quantum mechanics and structural chemistry. At the same time, they pointed out that the quantum theory of small molecules does not embrace all the possibilities for the immense mass of vital molecules that function in living things. A complete picture of life embraces the idea of dynamic energy landscapes, with dynamic conformations that are always interconverting between a variety of structures with varying energies, that is, undergoing state transitions. This idea recognizes that proteins are not rigid molecules. They exist as ensembles of states, which provide their physical foundation. Their distributions are described by the free energy landscape, which plots their conformational energy as a function of their conformational coordinates (del Sol et al. 2009; Kar et al. 2010; Kumar et al. 2000, 1999; Lu et al. 2016; Ma et al. 2000, 1999; Nussinov et al. 2023b, 2014, 2019a; Tsai et al. 1999, 2001; Tsai and Nussinov 2014). The sequences of the mutational variants are similar, implying that they likely share the same folds. However, their *preferred* conformational states (conformational coordinates) vary. The more extensive the cellular mutational load, the more heterogeneous the conformational ensemble. The mutations may be identical among the tumor cell population, or commonly differ, with the emergence of rare subclones. The affinities of the heterogeneous conformational ensemble to a prescribed drug vary, making cells that harbor rare mutations drug resistant.

Below, we discuss heterogeneous conformational ensembles within the framework of the free energy landscape followed by tumor cell populations. We then suggest how, considering tumor heterogeneity from the standpoint of conformational ensembles, can help clarify the behaviors and properties of populations of cells, including their phenotypes and plasticity, and provide a few cancer examples. Cell plasticity is an often-discussed rationale for cancer cells proliferation and resistance. We suggest that an apt description of plasticity could be in terms of dynamic ensembles, which better capture these properties, as offered by the Vonderheide and Stanger (Kim et al. 2024) and the Das, Tainer, and Pandita (Das et al. 2024) teams. Finally, we discuss heterogeneous conformational propensities of tumor cells as the daunting, foreboding drug resistance challenge, and how drug resistance and sensitive cell decimation, can further promote conformational diversity. On the *cellular* level (Xu et al. 2024), quantifying heterogeneity will need to consider single cell spatial biology, and its tumoral-interactions. On the *basic* level, the heterogeneity of the ensembles of spatially resolved cells could be quantified by their dynamic landscapes, capturing processes in disease. One challenge is how to translate the numbers into therapeutic regimen. One way is by selecting drug combinations targeting them (Gilad et al. 2021; Jaaks et al. 2022; Jin et al. 2023; Nair et al. 2023; Nussinov et al. 2024a, 2024c; Zhou et al. 2023), considering that intratumor heterogeneity can alter effective drugs in designed combinations observed by Lauffenburger and his colleagues over a decade ago (Zhao et al. 2014a). Below, we briefly overview the properties of conformational ensembles, important to understanding their connection to disease mechanisms.

## Biophysical properties of protein ensembles, cell plasticity, and proliferation

**Fig. 1:**
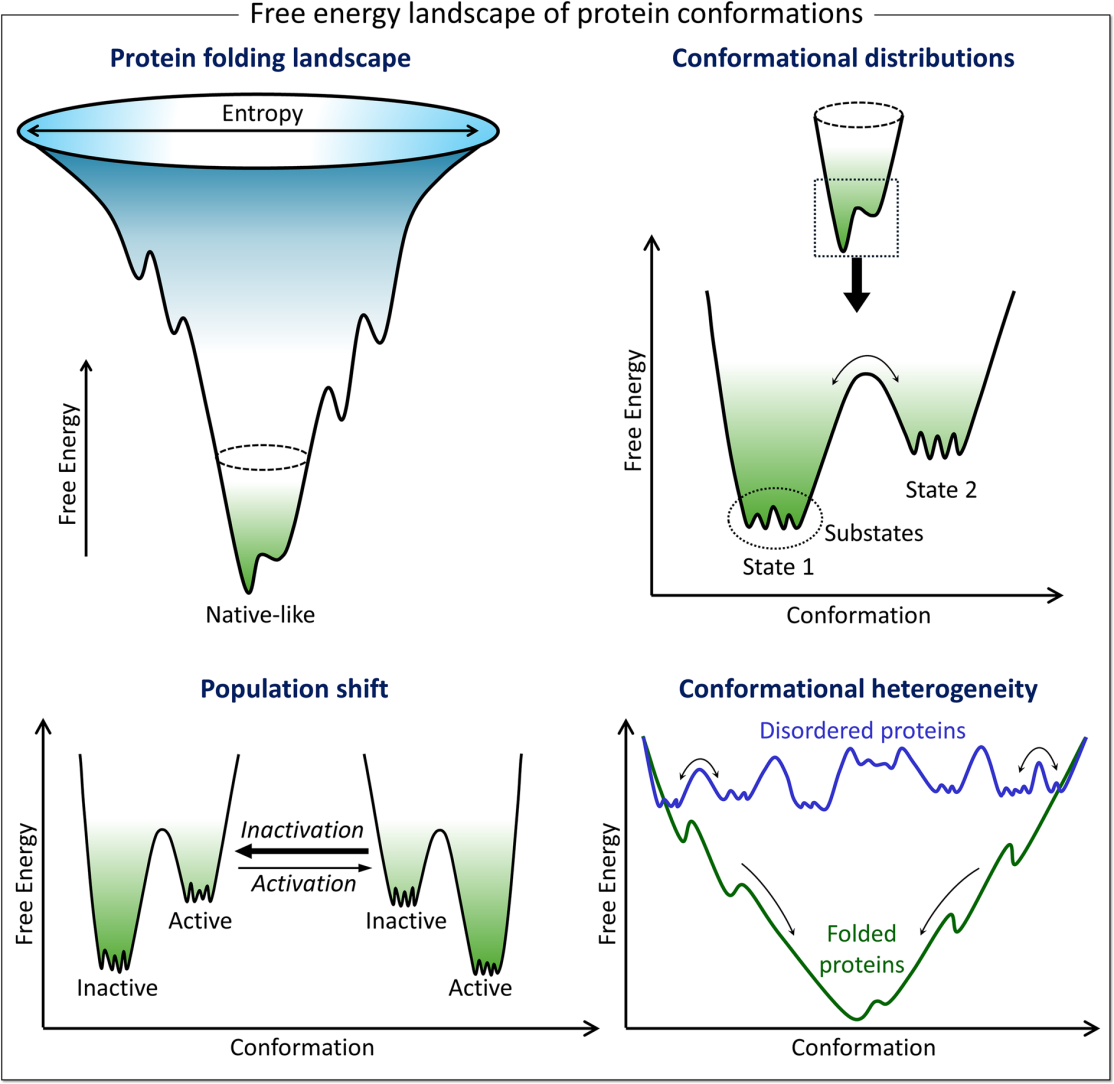
The ensembles of protein conformations. Funnel-shaped free energy landscape representing protein folding (top left). The width of the funnel represents the conformational entropy, and the local valleys and peaks represent the large ensembles of protein conformations. The native-like protein conformation denotes the lowest-energy valley at the bottom of the funnel. The conformational states separated by higher kinetic barriers correspond to different states of protein conformation (top right). In each well of the conformational states, proteins adopt multiple conformations with small differences in the substates separated by low kinetic barriers. The conformational distributions are influenced by the environment surrounding the proteins, including external factors such as mutations and posttranslational modifications (e.g., phosphorylation), which shift the population distribution of protein conformations (bottom left). For example, kinases typically occupy the inactive conformation under physiological conditions, and mutations can shift the population toward the active conformations. Examples of the heterogeneity of protein conformations shown in the folding free energy landscapes (bottom right). A folded protein has a free energy landscape with a well-defined global minimum, while a disordered protein has multiple minima in the free energy landscape, generating heterogeneous populations of conformational ensembles. Here, we do not discuss the disordered state. Instead, the heterogeneous populations are the outcome of mutations, variable expression, etc

The free energy landscape is complex (Baker et al. 2024; Frauenfelder et al. 1991; Wei et al. 2016; Yao and Hamelberg 2019). Around the native state, the conformational ensembles are in equilibrium, interconverting dynamically (Fig. 1). These dynamic state transitions hopping over kinetic barriers, which are not captured in the static landscapes (Frauenfelder et al. 1991), are the essence of function (Nagaraju et al. 2013). The conformational distributions are determined by statistical thermodynamics (Frauenfelder et al. 1991; Lyle et al. 2013), and the heights of the free energy barriers separating them define the conformational exchange rates. The distributions are influenced by their environments, including noncovalent binding, e.g., of substrates, membrane lipids (Akimoto et al. 2014; Gagne et al. 2015; Ishwar et al. 2015; Kloos et al. 2015; Roth and Bruggeman 2014; Rovira et al. 2015; Thompson et al. 2015), and covalent binding events, such as posttranslational modifications (e.g., phosphorylation), and crucial for our discussion here, mutations (Gao et al. 2009; Jimenez-Oses et al. 2014; Nussinov et al. 2012; Wei et al. 2016).

Proteins fluctuate (Iyer et al. 2021; Nussinov et al. 2023a). They visit states. The frequencies of the visits depend on the energy of the state; if high—it is rarely visited, if low it is frequent. Fluctuations of stable folded proteins trend toward homogeneous ensembles (Fig. 1). Fluctuations of intrinsically disordered, or unstable proteins generate heterogeneity (Nussinov et al. 2023b). The heterogeneity of cellular ensembles can be quantified by clustering (Kutlu et al. 2021), by assigning thermodynamic weights (Sayilgan et al. 2021), by cryo-EM/X-ray snapshots (Thomasen and Lindorff-Larsen 2022), NMR (Hansen et al. 2023), molecular dynamics (MD) simulations, and by MD/NMR combination (Abdelkarim et al. 2021; Jang et al. 2017), and other spectroscopic methods, such as FRET and DEER (Baker et al. 2024), all of which can provide their propensities.

The frequencies of the visits and the time that the conformations spend in the visited states are important, because they determine the heterogeneity of the ensemble and the cell phenotype. We suggested that a key parameter that decides cell proliferation is the number of conformations of the relevant oncogenic protein(s) in their active state. We defined propensities as that number. Knowledge of this number is consequential since it is a marker of the cell activity (Nussinov et al. 2023a). One way to measure cell activity is by quantitative conformational analysis, through structural features (Biddle et al. 2021; Khan et al. 2022). The relationship and significance of heterogeneous conformational ensembles for function have increasingly been considered (e.g., see Ding et al. 2023; Guzovsky et al. 2022; Horovitz et al. 2019, 2022; Kilinc et al. 2023; Kumar and Jernigan 2021; Nussinov et al. 2022a)). Since variants emerge during cancer evolution and drug resistance (Crucitta et al. 2022), the more diverse mutational and the expression loads, the more heterogeneous the ensembles, and the more heterogenous the cell populations. This clarifies the connection between mutational complexity, ensemble heterogeneity, and phenotypic manifestation (Alcolea et al. 2024).

Cancer aggressiveness is impacted by cellular plasticity. *Cellular plasticity* has been defined as the capacity of cells to adopt distinct identities within or across lineages (Perez-Gonzalez et al. 2023; Shen and Clairambault 2020), taking place during development, and cancer evolution, which harnesses embryonic developmental programs. We define plasticity in proteins and cells in terms of states (Nussinov et al. 2023a). A specific cell type embraces many cell states. *Cell type* has a specialized and fixed functionality. *Cell states* are influenced by the cell status, its environment, developmental state, etc. Transcriptomics can help identify a cell type; a specific cell type can exist in different states. A “protein state” relates to protein ensembles interconverting over time and environment. In the energy landscape, protein (sub)states located around the bottom of minima are separated by low kinetic barriers (Fig. 1). The ensemble of the protein (sub)states constitutes a “conformational state.” Conformational states are separated by higher barriers. We define cell state in terms of the Waddington landscape, where during their differentiation plastic cells can leap over mountains, overcoming barriers (Wang et al. 2011). Different cell states are more (less) pliable, with higher (lower) probabilities to transition into different cell types. In the Waddington probability landscape, different cell types correspond to different basins. Like the tumor heterogeneity, cellular plasticity in cancer cell populations is predicated on the dynamic fluctuations between states, however within or across lineages. Lineages in cancer proliferation require cell growth, pointing to alterations in chromatin accessibility and gene expression. In cancer, these are commonly the outcome of overexpression.

Overexpression is a hallmark of cancers. Below, we discuss the mechanisms through which cancers harness overexpression. In our heterogeneity context here, overexpression not only immensely increases the number of the active conformational states (Nussinov et al. 2022a, b). It also breaks feedback loops and homeostasis crosslinkages, breaching multiple oncogenic pathways (Gustin et al. 2009). Ongoing work aims to determine effective drug combinations to target key nodes co-populating proliferation pathways and their crosslinkages.

Collectively, the propensities of the conformations in the tumor cell population shape cell plasticity and tumor heterogeneity. When described in terms of dynamic changes (Ma et al. 1999; Tsai et al. 1999; Wei et al. 2016), conformational landscapes can capture enzyme catalysis and regulation (e.g., see Agback et al. 2023; Banerjee-Ghosh et al. 2020; Biddle et al. 2021; Blacklock and Verkhivker 2014; Boehr et al. 2009; Bunzel et al. 2021; Colizzi and Orozco 2021; Crawford and Sigman 2019; Cui and Karplus 2008; Damry et al. 2019; del Sol et al. 2009; Dennis 2022; Dixit and Verkhivker 2011; Gallagher et al. 2024; Gao and Klinman 2022; Gardner et al. 2020; Guillen-Pingarron et al. 2022; Guo and Zhou 2015; Ishiyama et al. 2023; Janson et al. 2023; Jones et al. 2024; Kamerlin and Warshel 2010; Kar et al. 2010; Khago et al. 2020; Kremer and Lyssiotis 2022; Leander et al. 2020; LeVine and Weinstein 2014; Lidbrink et al. 2024; Liu et al. 2022a; Ma and Nussinov 2013; Ma et al. 2011; Maria-Solano et al. 2018; Mathy and Kortemme 2023; McCullagh et al. 2024; Mingione et al. 2023; Nussinov et al. 2023b, 2014, 2013; Orellana 2019; Otten et al. 2018, 2020; Papaleo et al. 2016; Read et al. 2024; Reiss et al. 2020; Reiter et al. 2012; Rennella et al. 2023; Ruzmetov et al. 2024; Sasmal et al. 2024; Tsai et al. 2008; 2009; Verkhivker et al. 2020; Warshel and Bora 2016; Yabukarski et al. 2022; Zhang et al. 2022b)). Cancer embodies and exemplifies what can happen when these distributions are gone awry: heterogeneity, proliferation, and drug resistance.

## Linking tumor conformational states to disease mechanisms

Heterogeneity increases with tumor evolution. Heterogeneity can be within a tumor, between primary and secondary tumors, and between tumors of the same type in different patients. The varied (i) mutational load, (ii) relative expression of the protein(s) which are involved, and (iii) the cell’s microenvironment (Pietras and Sjolund 2024) are key determinants. For the first, the higher rate and incidence of cancer cell replication, a key hallmark of cell proliferation, set a higher likelihood of mutations, which vary across the tumor population. For the second, cancer cells harness diverse tactics to accomplish altered expression of certain cancer-related proteins (overexpression of oncogenes, under-expression of repressors), commonly involving structural changes in genomic regulatory regions, and especially, dysregulation of epigenetic modulators. Waddington’s diagrams capture the profound impact of the epigenetics landscape on cell fate (Ferrell 2012; Gilbert 1991; Noble 2015; Nussinov et al. 2024 d; Rajagopal and Stanger 2016; Waddington 1957), including how cells can adopt distinct identities during development, tissue homeostasis, and cancer evolution. As to the third, the microenvironment of cancer cells varies (Anderson and Simon 2020; de Visser and Joyce 2023). Cells excrete proteins and solutes. They also interact with, e.g., the tumor immune environment, which is also heterogeneous, including tumor-associated macrophages, and cancer-associated fibroblasts (CAFs). Their interactions can shift the relative stabilities of the ensembles.

The prime determinant of the cell phenotype is its transcriptome over time and the cell’s spatial position in the tumor and tissue (Morse et al. 2023; Nussinov et al. 2024 d; Walker et al. 2022; Williams et al. 2022; Zormpas et al. 2023). Changes in the expressed proteome—chiefly the outcome of epigenetic modifications—are responsible for the tumor heterogeneity. Dysregulation of expression can overflow inter-pathways connectors, aborting homeostasis. In cancer, the resultant altered proteomes trigger a larger population of the active protein states (Nussinov et al. 2024 d). The disproportional expression of the oncogenic states not only stimulate cancer growth and progression but also the emergence of multiple cancer subtypes co-existing in the same tumor, increasing drug resistance. One example is breast cancer, with biologically and clinically distinct subtypes. Current classification divides breast cancer into five groups, luminal A, luminal B, HER2-positive, basal-like, and normal breast-like (Eliyatkin et al. 2015). Claudin-low, another breast cancer subtype, is defined by gene expression characteristics. The claudin-low tumor has high expression of EMT genes. The histological stratification of breast cancers is based primarily on the expression of estrogen receptor (ER), progesterone receptor (PR), and HER2 (also known as ErbB2, encoded by *ERBB2*) (Table 1).

**Table 1:** Breast cancer subtypes based on molecular classification

Breast cancer subtypes	Hormone		Growth factor
	ER	PR	HER2
Luminal A	+	+	–
Luminal B	+	+	+
HER2-positive	–	–	+
Basal-like (TNBC)	–	–	–
Normal breast-like	+	+	–
Claudin-low	–	–	–

*ER* estrogen receptor, *PR* progesterone receptor, *HER2* human epidermal growth factor receptor type 2, *TNBC* triple-negative breast cancer

**Fig. 2:**
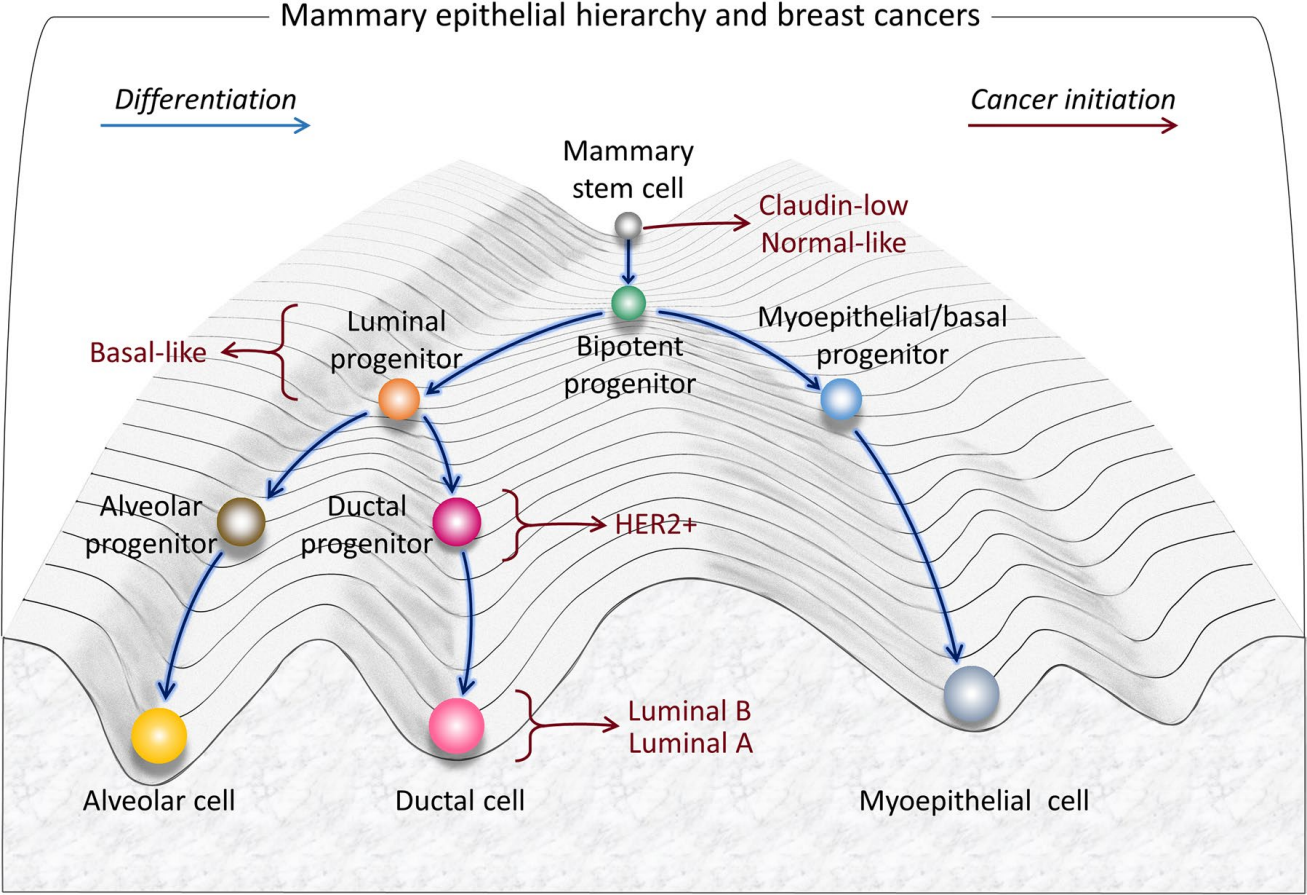
The model of mammary epithelial cell differentiation hierarchy based on the model of the epigenetic landscape described by Waddington. The multipotent mammary stem cell lineage proceeds downstream, differentiating into a bipotent progenitor, and unipotent cells such as luminal and myoepithelial progenitors. Alveolar, ductal, and myoepithelial cells are subtypes of these progenitors. Mutations in mammary stem cells can lead to abnormal differentiation, and transformation into cancer stem cells. Based on gene expression profiling (red arrows), breast cancer stem cells can develop into six breast cancer subtypes, which are linked to their closest normal epithelial counterparts. Claudin-low and normal breast-like subtypes have gene expression signatures similar to that of a mammary stem cell. The cell type origin of the basal-like subtype is bipotent progenitor or luminal progenitor. The HER2-positive subtype is associated with late luminal progenitors. The luminal A and luminal B subtypes are derived from differentiated luminal cells

In a pioneering work, Yeo and Guan described the multiple intratumoral co-existing breast cancer subtypes not only in terms of the groups and receptors which are involved but *cell state plasticity* (Yeo and Guan 2017). This work suggested that the dynamic conversions between the breast cancer types are the outcome of plasticity that captures the distinct differentiation states of the normal mammary gland hierarchy (Fig. 2). They suggested that cellular plasticity facilitates the transitions between subtypes within the same tumor, generating intra-tumor heterogeneity, which was further proposed could be detected by clustering the altered gene expression (Fougner et al. 2020; Pietras and Sjolund 2024; Yeo and Guan 2017). While not discussed there in this context, this view is reminiscent of Waddington’s embryonic cell lineage jumping over barriers on their way downhill, as can also be seen in the diagrams of the association between differentiation states of the mammary gland hierarchy and intrinsic breast cancer subtypes (Yeo and Guan 2017). In this view, the conjecture that the cancer stem cell (CSC) is in the least differentiated state has the highest tumorigenic potential is not universal. Normal cell lineage describes the developmental history of a cell into a specific cell type, or cell fate (Zenk et al. 2024).

The range of cellular phenotypes in tumors is jarring (Pe'er et al. 2021). Despite the traditional take on tumor heterogeneity resulting mainly from sequence alteration niches, current data indicate that the heterogeneity largely derives from epigenetic modulation, resulting from changes in the chromatin landscape. These changes involve opening relevant chromatin regions, which are condensed, or covered, in the non-transformed chromatin, making them transcriptionally available. As we discuss in the examples below, cancer adopts sweeping survival tactics, adopting multiple pro-life expression means, where sets of dozens of genes coordinate variability in their expression across malignant intratumor cells (Gavish et al. 2023). Chromatin-wise, all aim at altered expression levels, mostly of transcription factors and epigenetics modulators, receptor tyrosine kinases (RTKs), and other major oncogenic players, such as PI3K and B-Raf.

The emerging heterogenous cell populations do not morph into de novo states. Instead, they access earlier restricted states acting in embryonic developmental programs (Ciriello et al. 2024; Erdmann-Pham et al. 2023; Terekhanova et al. 2023). While unregulated cancer biology and regulated developmental biology appear to differ—in reality, embryos and cancers share multiple cellular and molecular features, including signaling pathways, cell migration processes and cell–cell interactions (Aiello and Stanger 2016). However, the cell states that they occupy differ from normal tissues.

## Cancer meta-tactics amass populations of active conformational states

**Fig. 3:**
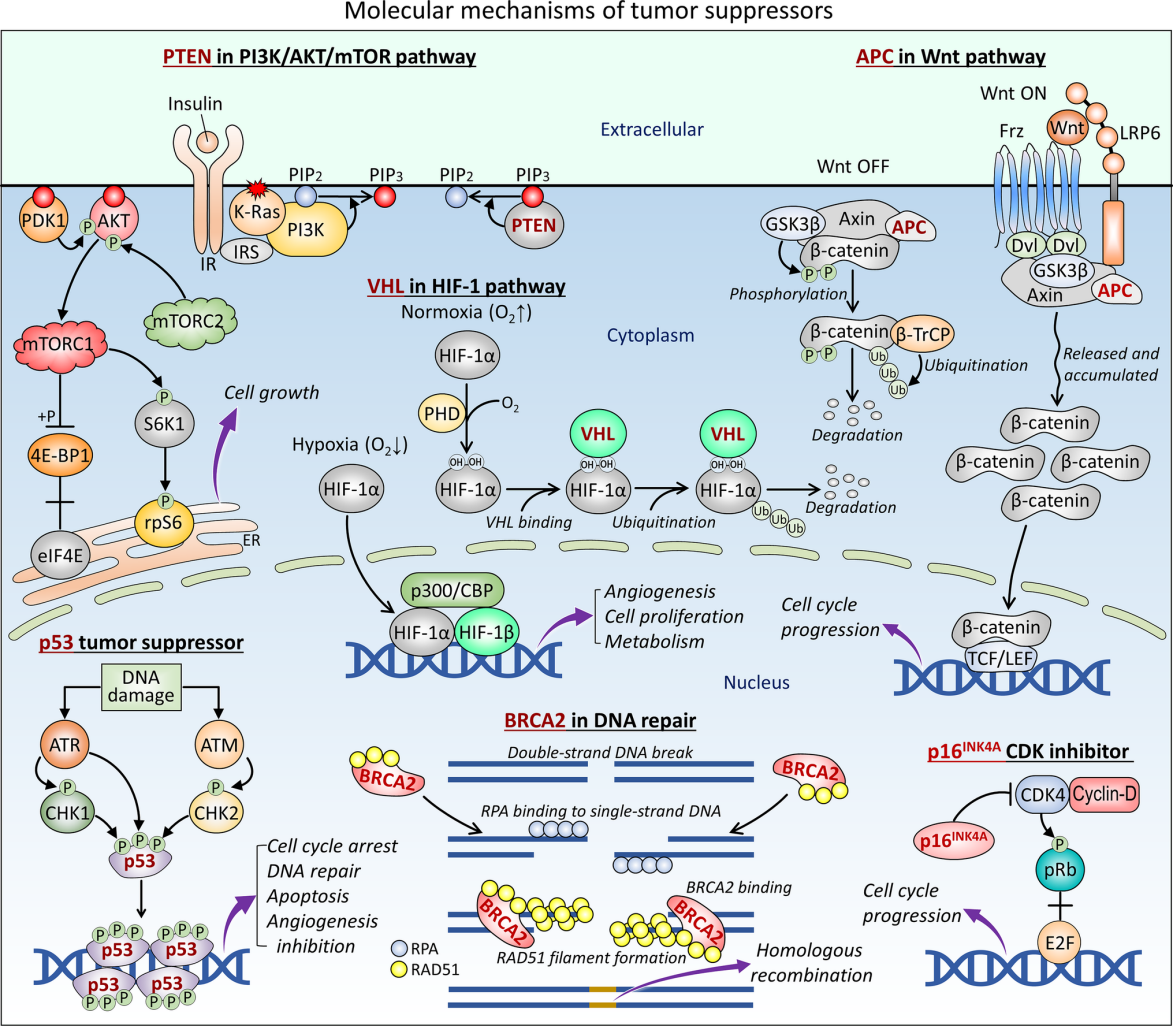
Molecular mechanisms of tumor suppressors. Here are examples for six tumor suppressors. (i) PTEN dephosphorylates PIP_3_ back to PIP_2_, which suppresses the IR-stimulated PI3K/AKT/mTOR pathway leading to cell growth. PI3K in complex with K-Ras and IRS phosphorylates PIP_2_ to PIP_3_, which recruits AKT, PDK1, and mTORC2 to the membrane. Active AKT is involved in the activation of mTORC1, which phosphorylates S6K1 and 4E-BP1. S6K1 activates rpS6. Phosphorylation of 4E-BP1 removes its inhibitory role on eIF4E, which is involved in cell growth. (ii) Wnt binds to Frz and LRP6, promoting Frz to recruit cytoplasmic Dvl, which prevents disruption of the APC/GSK3β/Axin complex and releases β-catenin. The released β-catenin accumulates and translocates to the nucleus where it activates the transcription factors TCF/LEF and promotes transcription. In the absence of Wnt, β-catenin is phosphorylated by GSK3β and degraded via ubiquitination by β-TrCP. (iii) In hypoxia with low oxygen levels, HIF-1α enters the nucleus and interacts with HIF-1β and the co-activators p300 and CBP to regulate cellular signaling. In normoxia with normal oxygen levels, HIF-1α is hydroxylated by PHD and recruits VHL, leading to ubiquitination and degradation. (iv) When DNA is damaged, ATR and ATM maintain the replication signaling pathway by activating CHK1 and CHK2, respectively. ATR, CHK1, and CHK2 phosphorylate p53, leading to p53 tetramerization in DNA, where p53 regulates anti-tumor signaling. (v) When double-strand DNA is broken, RPA coats single-strand DNA and promotes the interaction of the BRCA2 and RAD51 complex. RAD51 organizes into filaments that recruit sister DNA to replicate the damaged DNA by homologous recombination. (vi) In the G_1_ phase of the cell cycle, CDK4 activated by cyclin-D phosphorylates pRb, leading to its dissociation from the E2F transcription factor, which regulates cell cycle progression. The tumor suppressor p16 ^INK4 A^ inhibits CDK4. Abbreviations: PTEN, phosphatase and tensin homolog; IR, insulin receptor; PI3K, phosphoinositide 3-kinase; AKT, protein kinase B; mTORC, mammalian target of rapamycin complex; IRS, insulin receptor substrate; PDK1, 3-phosphoinositide-dependent protein kinase 1; Frz, frizzled; LRP6, low-density lipoprotein receptor-related protein 6; Dvl, dishevelled; APC, adenomatosis polyposis coli; GSK3β, glycogen synthase kinase 3β; axin, axis inhibition; TCF, “T cell” factor; LEF, lymphoid enhancer factor; HIF-1, hypoxia-inducible factor 1; CBP, CREB-binding protein; PHD, prolyl hydroxylase domain; VHL, von Hippel-Lindau; ATR, ATM and Rad3-related; ATM, ataxia-telangiectasia mutated; CHK1/2, checkpoint kinase 1/2; RPA, replication protein A; BRCA2, breast cancer type 2 susceptibility protein; RAD51, DNA repair protein RAD51 homolog 1; CDK4, cyclin-dependent kinase 4

“Meta” has been taken as designating the most effective tactics available. Judging by the tactics used across the various cancers, this definition fits well. Cancers’ meta-tactics are diverse. Nonetheless, they share a common feature: harnessing activating mutations and over- (under-) expression levels, with both working through a change in the number of active conformational states. The main contributor are cancer-related genes. Classically, genetic mutations in oncogenes were taken as the main cancer sponsor, coupled with genetically tamped down actions by cancer suppressors, such as p53, INK4, PTEN, VHL, APC, BRCA2, and pRb (Fig. 3). This view was nurtured by the discoveries of the many driver mutations through large-scale sequencing. However, accumulating data over the last few years update this view. Whereas driver mutations are a vastly important factor—as can be seen in the large statistics and correlations with cancer—changes in expression levels via epigenetics appear the dominant agent.

**Fig. 4:**
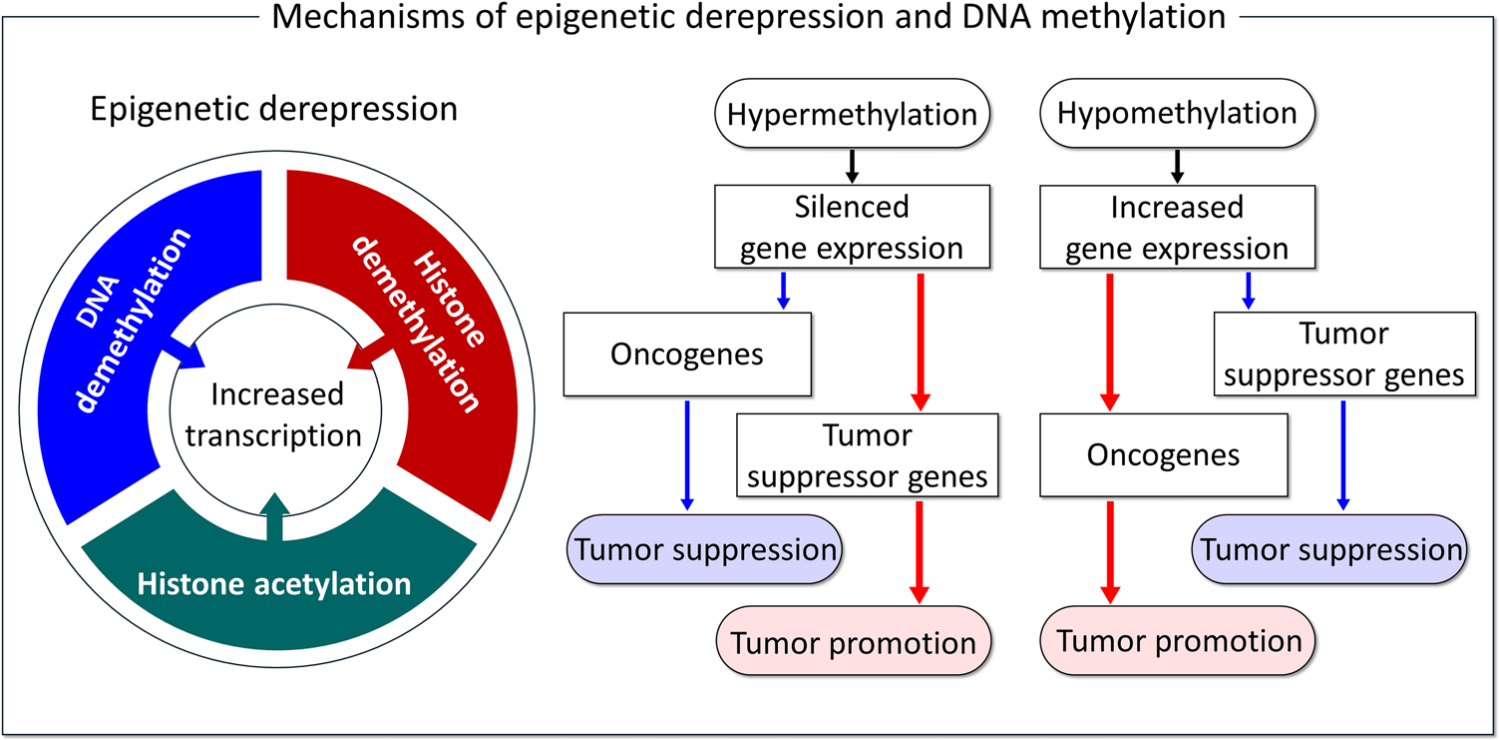
Mechanisms of epigenetic derepression and DNA methylation. A schematic diagram representing epigenetic derepression caused by DNA and histone demethylations and histone acetylation (left panel). DNA methylation is a chemical modification that adds methyl groups to DNA. As a well-characterized epigenetic modification, it controls gene expression and genome stability. Loss of DNA methylation causes a variety of diseases, including cancer. Both DNA hypermethylation and hypomethylation occur in cancer. Hypermethylation on tumor suppressor genes and hypomethylation on oncogenes can lead to cancer (right panel)

Expression of oncogenes or cancer-promoting genes can be extremely high through epigenetic derepression mechanisms (Fig. 4), involving, e.g., DNA and histone demethylations (Jeong et al. 2014) and histone acetylation (Martin et al. 2021; Sterner and Berger 2000), all increase transcription by making DNA more accessible to transcription factors (Lee et al. 2020b). Expression of tumor suppressor genes can be low because of methylation and deacetylation. Hypermethylation can silence gene expression, especially tumor suppressor genes, as in the case of ovarian cancer, where it is a poor prognosis factor (Feng et al. 2019). Hypomethylation is likely to have an increased potential for expression and serves as a possible epigenetic mechanism promoting tumorigenesis. Aberrant DNA methylation is common. Another example involves neuroblastoma, a pediatric cancer of the sympathetic nervous system and one of the most common pediatric solid tumors (Fetahu and Taschner-Mandl 2021). Its epigenetic alterations include aberrant DNA methylation, histone modifications, alterations in chromatin composition and organization, and expression of non-coding RNAs. Its methylation status can predict survival (Lalchungnunga et al. 2022). MicroRNAs and alternative splicing are also emerging among the regulators of epigenetic mechanisms. In addition to epigenetic derepression, gene amplification can also lead to overexpression of genes (Vernimmen et al. 2003), as in neuroblastoma (Fetahu and Taschner-Mandl 2021), through *MYCN* copy number alterations, and genomic rearrangements (Banerjee et al. 2024b). Histone hyperacetylation disrupts the gene regulatory architecture in pediatric rhabdomyosarcoma (Gryder et al. 2019), a highly aggressive cancer that develops from mesenchymal cells that have failed to fully differentiate into myocytes of skeletal muscle.

The *ERBB2* gene in breast cancer is another prime example of overexpression. *ERBB2* is overexpressed in 30% of breast cancers. It is also upregulated in, e.g., prostate, pancreas, colon, and ovary cancers. In breast cancer cells, the mechanism leading to *ERBB2* gene overexpression is gene amplification, including copy number (Dumbrava et al. 2019; Ellegard et al. 2019; Harari and Yarden 2000). HER2 (or ErbB2) amplification results in hyperactivation of a signaling network, dysregulating the G_1_/S checkpoint through high levels of active CDK4/6 in complex with cyclin-D (Harari and Yarden 2000). In breast cancer, HER2 can be increased by 40 ~ 100-fold and the gene can be expressed at 25 ~ 50-fold (Galogre et al. 2023).

In lung adenocarcinoma, *ERBB2* overexpression was also observed to take place through super-enhancer, where multiple transcription factors bind multiple enhancers to drive transcription (Kaneko et al. 2024). Additional genes with significant amplification of expression exploit copy number changes (Beroukhim et al. 2010; Zack et al. 2013), e.g., of Myc transcription factor in pancreatic cancer (Oketch et al. 2023), and diffuse large B-cell and high-grade lymphoma (Schieppati et al. 2020), and *MYC* and *TERT* copy number variations in lung cancer (Brik et al. 2023). These amplified copy numbers may overlap the super-enhancers and structural, further driving gene expression (Kaneko et al. 2024).

**Fig. 5:**
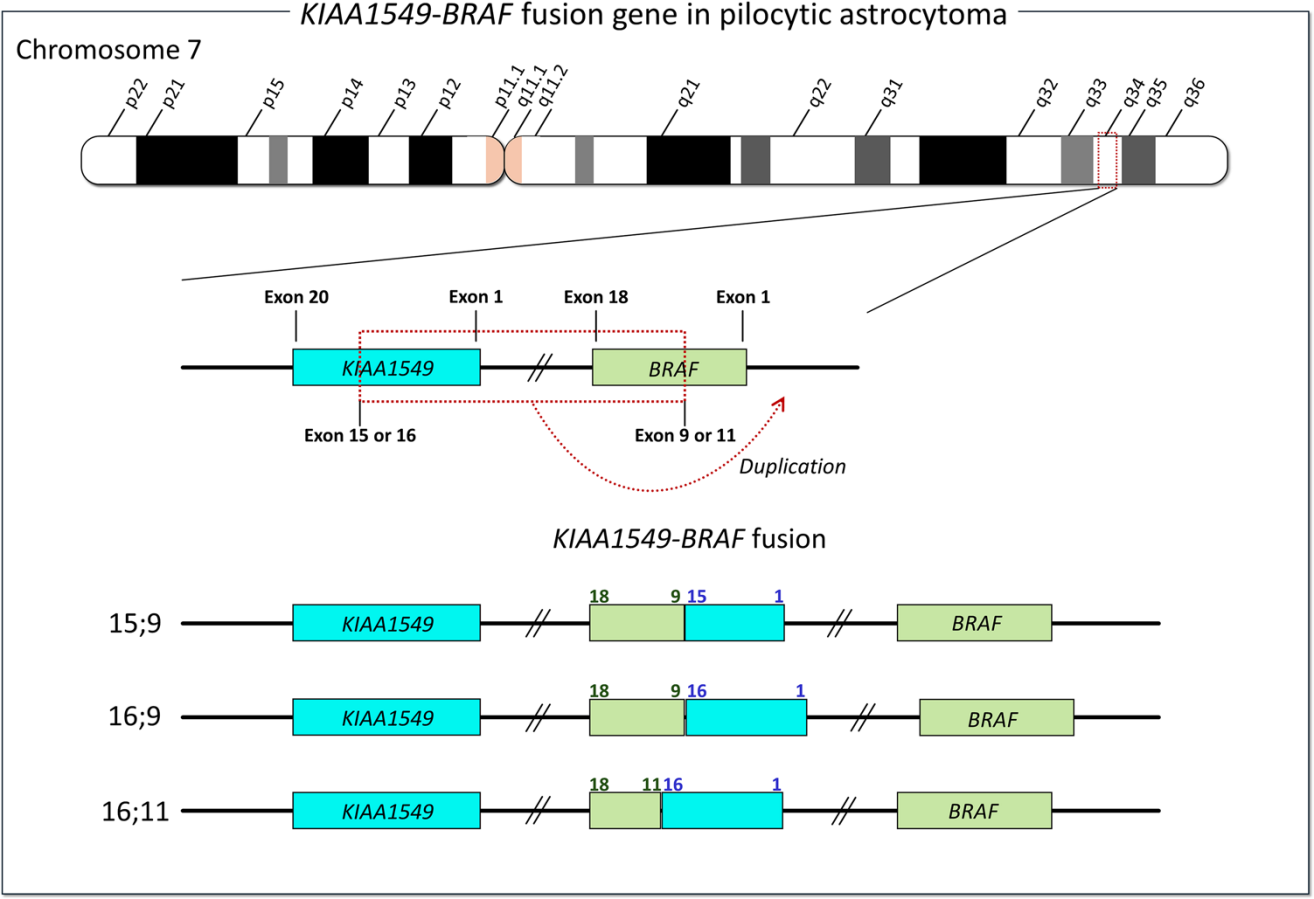
*KIAA1549*-*BRAF* fusion gene in pilocytic astrocytoma. Both *KIAA1549* and *BRAF* genes are located on the q34 band of chromosome 7. Duplication of *KIAA1549* with exon 1 to exon 15 or exon 16 and *BRAF* with exon 9 or exon 11 to exon 18 results in the *KIAA1549*-*BRAF* fusion gene. This fusion gene encodes a chimeric protein with the N-terminal truncated B-Raf (i.e., kinase domain only), which has a high affinity for dimerization and activation

A major additional cancer tactic is chromosome rearrangements, including fusion events (Pierotti et al. 2003). Fusion genes can involve joining segments from two different genes commonly the head of one gene and the tail of another, with both genes contributing to the transforming potential of the chimeric oncoprotein. *BRAF* gene fusions in solid tumors are frequent (Ross et al. 2016), in addition to high frequencies of some B-Raf mutations, such as B-Raf^V600E^. *BRAF* fusions are predominantly in gliomas, including glioblastoma. Notably, they are also observed in melanoma (Botton et al. 2019), and other cancers. The fusions involve diverse genes, such as the *KIAA1549*-*BRAF* fusion gene in pilocytic astrocytoma (Fig. 5) (Appay et al. 2018; Hawkins et al. 2011; Krynina et al. 2024), a pediatric brain tumor, which demonstrates significant structural diversity. The oncogenic potential of *BRAF* is commonly due to the loss of B-Raf’s N-terminal domains, which are critical for its autoinhibition (Maloney et al. 2022; Weinberg et al. 2020; Zhang et al. 2021).

Finally, there is the 2007 observation (Axelsen et al. 2007) that cancers overexpress only a few genes that are expressed in the tissue in which the cancer originated. Within this framework, melanomas overexpressed the highest number of brain-selective genes, which may explain why melanoma metastasizes to the brain.

Altogether, similar programs exist across cancer types (Baron et al. 2020; Gavish et al. 2023; Kinker et al. 2020), suggesting that they are related to basic tumor biology. High expression through a meta-tactic can define a cell state—which is influenced by its heterogeneous conformational ensemble.

## Conclusions

The mechanisms for observations such as the melanoma above (Axelsen et al. 2007) is not fully understood, nor are the mechanisms explaining exactly how metastatic breast, lung, and metastatic melanoma tumors achieve vastly different somatic mutation profiles from the AML. Thirty-two mutations were identified in breast cancer—and 33,345 and 22,910 in the melanoma and lung cell lines (Harjes 2020), respectively. Further, mutations identified breast tumors were largely mutually exclusive from those in 192 AML tumor genomes. Large-scale studies such as The Cancer Genome Atlas (TCGA) and the Pan-Cancer Analysis of Whole Genomes (PCAWG) have now sequenced tens of thousands of cancer genomes across a wide variety of tumor types, providing huge amount of data. Carried out over spatially distinct cells, over time, and coupled with transcriptomes, proteomes, and clinical data, unveil additional levels of heterogeneity—but still lacking the mechanisms at the basic molecular behavior.

The basic physical sciences can step in. Beyond the development of quantitative techniques and tools, they can help establish conceptual frameworks with foundational contributions. These could guide significant advances in understanding the disease, and by integrating whole-genome sequencing, profiles of transcriptomes and epigenomes, gain deeper understanding of the cancers’ complex tactics. Cancer has been described as “a complex dynamic system in which evolving cells both affect and are affected by the physical properties of their environment” (White et al. 2019), leading to the NCI’s Physical Sciences—Oncology Network (PS-ON) initiative “to foster the convergence of physical sciences approaches and perspectives with cancer research to advance our understanding of oncology.” Especially, these could permit making better, patient-specific, pharmacological predictions.

The prime determinant of the cell phenotype is its transcriptome, which varies over time and the cell’s spatial position (Morse et al. 2023; Nussinov et al. 2024 d; Walker et al. 2022; Williams et al. 2022; Zormpas et al. 2023), making single cell spatial biology key factor in tumor heterogeneity. At the same time, the behavior of single cells within the tumor is decided by their interactions and their transcriptomes, read and translated, by their conformational ensembles. We believe that tumor heterogeneity is not random, as observed in metastasis trends. Studies of *heterogeneous cell populations* emerging in cancer tissues from diverse cell types could inform the likely heterogeneous populations, which could guide drug combinations. Cell phenotypes could be learned from *conformational ensembles* (Nussinov et al. 2025).

## Data Availability

No datasets were generated or analysed during the current study.
